# Impact of COVID‐19 on the incidence of localized and metastatic prostate cancer among White and Black Veterans

**DOI:** 10.1002/cam4.5151

**Published:** 2022-08-19

**Authors:** Kyung Min Lee, Alex K. Bryant, Patrick Alba, Tori Anglin, Brian Robison, Brent S. Rose, Julie A. Lynch

**Affiliations:** ^1^ VA Informatics and Computing Infrastructure VA Salt Lake City Health Care System Salt Lake City Utah USA; ^2^ Department of Radiation Oncology Veterans Affairs Ann Arbor Healthcare System Ann Arbor Michigan USA; ^3^ Department of Radiation Oncology University of Michigan Ann Arbor Michigan USA; ^4^ VA San Diego Healthcare System San Diego California USA; ^5^ Department of Radiation Medicine and Applied Sciences University of California, San Diego La Jolla California USA; ^6^ Department of Urology University of California, San Diego La Jolla California USA

**Keywords:** cancer screening, COVID‐19, incidence of prostate cancer, racial disparities, veterans affairs

## Abstract

The COVID‐19 pandemic disrupted prostate‐specific antigen (PSA) screening and prostate biopsy procedures. We sought to determine whether delayed screening and diagnostic workup of prostate cancer (PCa) was associated with increased subsequent rates of incident PCa and advanced PCa and whether the rates differed by race. We analyzed data from the Veterans Health Administration to assess the changes in the rates of PSA screening, prostate biopsy procedure, incident PCa, PCa with high‐grade Gleason score, and incident metastatic prostate cancer (mPCa) before and after January 2020. While the late pandemic mPCa rate among White Veterans was comparable to the pre‐pandemic rate (5.4 pre‐pandemic vs 5.2 late‐pandemic, *p* = 0.67), we observed a significant increase in incident mPCa cases among Black Veterans in the late pandemic period (8.1 pre‐pandemic vs 11.3 late‐pandemic, *p* < 0.001). Further investigation is warranted to fully understand the impact of the COVID‐19 pandemic on the diagnosis of advanced prostate cancer.

## Graphical abstract

The COVID‐19 pandemic disrupted prostate‐specific antigen (PSA) screening and subsequent prostate biopsy procedures. In this analysis, Black Veterans appeared to have experienced a late‐pandemic rise in mPCa incidence not observed among White Veterans. " cd_value_code="text

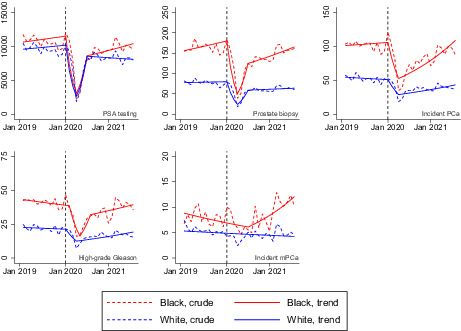

## 1 INTRODUCTION

Early‐stage prostate cancer (PCa) is typically detected on prostate‐specific antigen (PSA) screening and subsequent prostate biopsy. The COVID‐19 pandemic disrupted such routine healthcare screenings and interventions^1^, ^2^ and may have had a larger effect on Black men, who are at a higher baseline risk of both localized and metastatic PCa (mPCa).^3^ We sought to determine whether delayed screening and diagnostic workup of PCa was associated with increased subsequent rates of incident PCa (localized or metastatic), PCa with high‐grade Gleason score, and incident mPCa and whether the rates differed by race.

## 2 METHODS

We analyzed PSA testing, prostate biopsy, and PCa diagnosis data from January 2019 to August 2021 obtained from the Corporate Data Warehouse of the Veterans Health Administration. The study sample included all male Veterans aged 40 years and older with a self‐reported race of either non‐Hispanic White (White) or Black. For each race, we calculated monthly rates of PSA testing, prostate biopsy, incident PCa diagnosis, PCa with high‐grade Gleason score (defined as a Gleason 4 + 3 or higher), and incident mPCa by dividing the number of unique patients who underwent the procedure or were diagnosed in each month by the average number of men aged 40 or older who received care (inpatient admission or outpatient visit) in the same month from 2016 to 2018. We used the average of the 3 years prior to the pandemic as the denominator to obtain a more stable denominator unaffected by the pandemic. Incident PCa was defined by a first International Classification of Disease, Tenth Revision, Clinical Modification code for PCa accompanied by documentation of a Gleason score or metastatic disease within +/− 90 days, ascertained by a validated natural language processing tool.^4^ We plotted monthly crude rates per 100,000 men by race before and after January 2020, the month in which the U.S. declared COVID‐19 a public health emergency.^5^ We divided the pandemic period into two segments: the early pandemic (February 2020 to February 2021) and the late pandemic (March 2021 to August 2021). We performed two‐tailed t‐tests to compare the mean rates of PSA testing, prostate biopsy, incident PCa, PCa with high‐grade Gleason score, and incident mPCa from January 2019 to January 2020 (pre‐pandemic) to those in the early pandemic and the late pandemic periods within each race. As a secondary analysis, we performed join point regression analysis to identify significant trends in the crude rates of PSA testing, prostate biopsy, incident PCa, PCa with high‐grade Gleason score, and incident mPCa during the study period.^6^ All data preparation and statistical tests were performed using SAS 9.2 (Cary, NC); join point regression analysis was performed using Joinpoint Regression Program, Version 4.9.1.0.^7^

## 3 RESULTS

**FIGURE 1:**
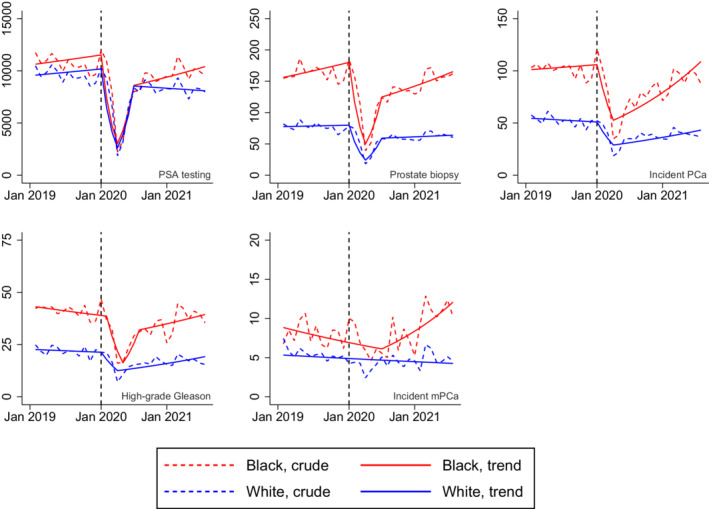
Monthly rates of prostate‐specific antigen testing, prostate biopsy, incident prostate cancer diagnosis, high‐grade Gleason score, and incident metastatic disease per 100,000 men by race. Denoted by a vertical dashed line in each of the five panels, January 2020 was the month in which the U.S. Department of Health and Human Services declared COVID‐19 a public health emergency in the U.S. mPCa, metastatic prostate cancer; PCa, prostate cancer; PSA, prostate‐specific antigen.

Black Veterans had higher pre‐pandemic rates of PSA testing, prostate biopsy, incident PCa, and incident PCa with high‐grade Gleason score compared to White Veterans (Figure 1 and Table 1). The rates decreased substantially in both Blacks and Whites from February 2020 to April 2020 before recovering to pre‐pandemic levels in subsequent months (Figure). The absolute declines in PSA testing, prostate biopsy, and incident PCa diagnoses were larger among Black Veterans compared to those of White Veterans. While the late pandemic mPCa rate among White Veterans was comparable to the pre‐pandemic rate (5.4 pre‐pandemic vs 5.2 late‐pandemic, *p* = 0.67), we observed a significant increase in incident mPCa cases among Black Veterans in the late pandemic period (8.1 pre‐pandemic vs 11.3 late‐pandemic, *p* < 0.001; Table). Join point analysis results showed that, while there was no significant trend in mPCa rate among White Veterans, mPCa cases among Black Veterans significantly increased by 5.4% monthly from July 2020 to August 2021 (Table S1).

**TABLE 1:** Mean rates per 100,000 men of prostate cancer‐related measures before and after COVID‐19 by race.

	PSA screening rate		Prostate biopsy rate		Incident PCa rate		Incident PCa with high‐grade Gleason rate		Incident mPCa rate	
	Black	White	Black	White	Black	White	Black	White	Black	White
Pre‐pandemic, mean (*SD*)^a^	10,735.1 (792.5)	9601.9 (642.2)	164.0 (12.7)	77.0 (6.1)	102.3 (8.3)	52.3 (4.7)	41.3 (3.1)	21.8 (2.3)	8.1 (1.5)	5.4 (0.8)
Early pandemic, mean (*SD*)^b^	7964.3 (2571.9)	7254.8 (2254.)	117.4 (34.6)	53.1 (14.8)	71.6 (18.7)	35.1 (7.7)	29.0 (6.9)	15.4 (3.5)	6.9 (1.9)	4.2 (0.8)
Late pandemic, mean (*SD*)^c^	10,122.0 (768.5)	8268.5 (662.5)	161.3 (8.1)	65.4 (4.2)	95.6 (6.1)	40.2 (3.3)	40.0 (3.4)	17.7 (1.8)	11.3 (1.1)	5.2 (1.0)
Difference in mean rates, early pandemic vs. pre‐pandemic (*p*‐value)^d^	−2770.8 (*p* = 0.001)	‐2347.1 (*p* = 0.001)	−46.5 (*p* < 0.001)	−23.9 (*p* < 0.001)	−30.6 (*p* < 0.001)	−17.2 (*p* < 0.001)	−12.3 (*p* < 0.001)	−6.5 (*p* < 0.001)	−1.2 (*p* = 0.09)	−1.3 (*p* < 0.001)
Difference in mean rates, late pandemic vs. pre‐pandemic (*p*‐value)	−613.1 (*p* = 0.13)	−1333.4 (*p* < 0.001)	−2.7 (*p* = 0.65)	−11.6 (*p* < 0.001)	−6.7 (*p* = 0.09)	−12.2 (*p* < 0.001)	−1.3 (*p* = 0.42)	−4.2 (*p* = 0.001)	3.2 (*p* < 0.001)	−0.2 (*p* = 0.67)

Abbreviations: mPCa, metastatic prostate cancer; PCa, prostate cancer; PSA, prostate‐specific antigen; *SD*, standard deviation.

^a^ January 2019–January 2020.

^b^ February 2020–February 2021.

^c^ March 2021–August 2021.

^d^ *P*‐values were generated by two‐tailed *t*‐tests comparing mean monthly rates in each era (pre‐pandemic, early pandemic, and late pandemic).

## 4 DISCUSSION

In this analysis of the nationwide Veterans Affairs Healthcare System, we found that the rates of PSA screening, prostate biopsy, and incident PCa diagnosis decreased substantially in the early phase of the COVID‐19 pandemic, with larger absolute declines observed among Black Veterans compared to White Veterans. Black Veterans appeared to have experienced a late‐pandemic rise in mPCa incidence not observed among White Veterans.

Other studies have similarly shown dramatic declines in cancer screening procedures and PSA screening rates,^1^, ^2^, ^8^ though none have examined recent changes in incident mPCa rates. Leveraging a natural language processing tool that allows rapid identification of incident mPCa cases,^4^ we found an increase in mPCa incidence among Black Veterans in the late‐pandemic period. This may be related to the higher baseline risk of incident mPCa and larger absolute declines in screening tests and procedures among Black compared to White Veterans leading to delayed diagnoses. A literature review has shown that multiple factors contributed to substantial reductions in PSA screening and prostate biopsy rates, including physician reassignment, institutional, and government policies, and patients' avoidance of health care interaction out of fear that they might contract severe acute respiratory syndrome coronavirus 2.^9^ Based on their analysis of the Medicare claims data, Patt et al.^8^ have suggested that the decline in cancer screening in conjunction with decreased in‐person visits and cancer‐related procedures may result in a stage migration to more advanced cancer at diagnosis. Further investigation is warranted to fully understand the impact of the COVID‐19 pandemic on the diagnosis of advanced prostate cancer.

## AUTHOR CONTRIBUTIONS

Kyung Min Lee: Data curation, study design, data analysis and interpretation, writing—drafting, review, and editing. Alex K. Bryant: Data analysis and interpretation, writing—drafting, review, and editing. Patrick Alba: Methodology, writing—review and editing. Tori Anglin: Data collection. Brian Robison: Data collection. Brent S. Rose: Conceptualization, study design, data interpretation, writing—review and editing. Julie A. Lynch: Conceptualization, data interpretation, writing—review and editing.

## CONFLICT OF INTEREST

The authors declare no competing financial interests.

## ETHICS STATEMENT

This study was approved by the University of Utah Institutional Review Board and VA Salt Lake City Research & Development committee. Analyses were conducted under the approved waiver of informed consent and HIPAA authorization.

## Supporting information


Table S1

## Data Availability

The Corporate Data Warehouse of the Veterans Health Administration analyzed in this study is not publicly available.
