# Supplementary material for: Impact of COVID‐19 on the incidence of localized and metastatic prostate cancer among White and Black Veterans
Source: Cancer Med. 2022 Aug 19;12(3):3727–30. doi: 10.1002/cam4.5151 (PMC9538539; doi:10.1002/cam4.5151)
Supplement: Supplementary file 1 — Table S1 [file CAM4-12-3727-s001.docx]

**Supplementary Table**. Trends in monthly rates per 100,000 men of prostate-specific antigen screening, prostate biopsy, incident prostate cancer, and high-grade Gleason score, and metastatic disease at diagnosis by race

|  | Black | | | White | | |
| --- | --- | --- | --- | --- | --- | --- |
|  | MPC | 95% C.I. | | MPC | 95% C.I. | |
| PSA testing |  |  |  |  |  |  |
| 1/2019 - 1/2020 | 0.7 | -0.4 | 1.8 | 0.5 | -0.5 | 1.5 |
| 1/2020 - 4/2020 | -35.8 | -46.7 | -22.6 | -36.3 | -46.3 | -24.5 |
| 4/2020 - 7/2020 | 41.3 | 17.3 | 70.3 | 48.2 | 25.0 | 75.6 |
| 7/2020 - 8/2021 | 1.5 | 0.5 | 2.5 | -0.4 | -1.3 | 0.5 |
| Prostate biopsy |  |  |  |  |  |  |
| 1/2019 - 1/2020 | 1.2 | -0.1 | 2.5 | 0.2 | -1.1 | 1.6 |
| 1/2020 - 4/2020 | -34.8 | -47.9 | -18.5 | -32.9 | -46.5 | -15.9 |
| 4/2020 - 7/2020 | 35.8 | 8.6 | 69.9 | 35.0 | 7.6 | 69.2 |
| 7/2020 - 8/2021 | 2.2 | 1.0 | 3.4 | 0.6 | -0.6 | 1.8 |
| Incident PCa |  |  |  |  |  |  |
| 1/2019 - 1/2020 | 0.4 | -1.8 | 2.6 | -0.6 | -2.5 | 1.5 |
| 1/2020 - 4/2020 | -20.8 | -45.1 | 14.3 | -17.2 | -41.2 | 16.6 |
| 4/2020 - 8/2021 | 4.7 | 3.2 | 6.1 | 2.5 | 1.2 | 3.9 |
| High-grade Gleason |  |  |  |  |  |  |
| 1/2019 - 2/2020 | -0.8 | -2.6 | 1.0 |  |  |  |
| 2/2020 - 5/2020 | -25.0 | -46.8 | 5.7 |  |  |  |
| 5/2020 - 8/2020 | 25.3 | -11.1 | 76.7 |  |  |  |
| 8/2020 - 8/2021 | 1.7 | -0.3 | 3.8 |  |  |  |
| 1/2019 - 1/2020 |  |  |  | -0.5 | -2.5 | 1.6 |
| 1/2020 - 4/2020 |  |  |  | -16.3 | -41.1 | 19.0 |
| 4/2020 - 8/2021 |  |  |  | 2.7 | 1.4 | 4.1 |
| mPCa |  |  |  |  |  |  |
| 1/2019 - 7/2020 | -2.0 | -4.1 | 0.1 |  |  |  |
| 7/2020 - 8/2021 | 5.4 | 1.8 | 9.1 |  |  |  |
| 1/2019 - 8/2021 |  |  |  | -0.7 | -1.5 | 0.1 |

MPC: monthly percent change; C.I.: confidence interval; PSA: prostate-specific antigen; PCa: prostate cancer; mPCa: metastatic prostate cancer
